# Development of High-Efficiency Fertilizer by Hydrogels Obtained from Cassava Starch and Citric Acid for Slow Release of Ammonium and Potassium

**DOI:** 10.3390/gels10070434

**Published:** 2024-06-29

**Authors:** Andrés F. Chamorro, Manuel Palencia, Álvaro A. Arrieta

**Affiliations:** 1 Research Group of Electrochemistry and Environment (GIEMA), Faculty of Basic Sciences, Universidad Santiago de Cali, Cali 760035, Colombia; 2 Research Group in Science with Technological Applications (GICAT), Department of Chemistry, Faculty of Natural and Exact Science, Universidad del Valle, Cali 760032, Colombia; 3 Department of Biology and Chemistry, Faculty of Education and Sciences, Universidad de Sucre, Sincelejo 700003, Colombia; alvaro.arrieta@unisucre.edu.co

**Keywords:** cassava starch, hydrogel, high-efficiency fertilizers, sustainable agriculture

## Abstract

Fertilizers with enhanced efficiency or high-efficiency fertilizers increase the nutrient availability, minimize losses, and reduce costs, thereby increasing crop yields and food production while mitigating environmental impacts. This research evaluates the synthesis of biodegradable hydrogels from cassava starch and citric acid for agrochemical applications. Hydrogels were synthesized using water as the solvent and applied for the controlled release of macronutrients (N and K). Four concentrations of nutrient-containing salts were tested (0.5 to 10.0% w/w). Materials were analyzed using ATR-FTIR spectroscopy and swelling studies. The presence of nutrients reduced both the crosslinking efficacy and the water absorption capacity, with the latter dropping from 183.4 ± 0.6% to 117.9 ± 3.7% and 157.4 ± 25.0% for hydrogels loaded with NH_4_Cl and KCl, respectively. The cumulative release of K and N from the hydrogel was monitored for 144 h and examined using kinetics models, revealing that the releases follow Fickian’s diffusion and anomalous diffusion, respectively. Additionally, the material was formed using cassava with peel previously milled to reduce the production costs, and its potential for nutrient-controlled delivery was evaluated, with the finding that this hydrogel decreases the release rate of nitrogen. The results suggest that these biomaterials may have promising applications in the agrochemical industry in the making of high-efficiency fertilizers.

## 1. Introduction

The continuous rise in global food demand requires an increase of approximately 60% in agricultural production to feed an estimated 9.7 billion people by 2050 [1]. Traditionally, fertilizers have significantly increased crop yields over the last 50 years. However, the excessive and inefficient use of fertilizers has revealed several drawbacks, including economic losses, increased food prices, and environmental contamination [2,3]. Currently, fertilizers exhibit poor nutrient use efficiency due to the loss of many nutrients thought various processes such as leaching, runoff, volatilization, and lixiviation [3,4]. These processes result in economic losses for agricultural producers and an increase in the cost of food. Additionally, the loss of nutrients contributes to the environmental contamination of water, soil, and air and can even impact human health. For instance, the nitrogen use efficiency from urea is approximately 40% [5], indicating that more than 50% of the applied nitrogen is lost and transported into the environment. This leads to the formation of nitrite in water, which reduces oxygen levels and hinders the development of aquatic species. Nevertheless, a new type of fertilizer has been proposed as an alternative to overcome the limitations of conventional fertilizers. These are the called “fertilizers with enhanced efficiency” or “high-efficiency fertilizers”. These products are designed to increase nutrient availability, minimize losses, and reduce the costs of agricultural production. Consequently, their objective is to increase crop yields, enhance food production, and reduce the negative environmental impacts of agriculture. Among the most promising matrices for these fertilizers are polymeric hydrogels, particularly those of natural origin (biopolymeric hydrogels).

Polymeric hydrogels are three-dimensional structures with crosslinking, characterized by their high water absorption capacity (WAC), low solubility, high durability, and adequate stability. These materials have demonstrated their ability to reduce soil erosion and runoff by altering the hydrophysical properties of soil [6,7]. Furthermore, they can trap nutrients and promote their slow release in soils. Synthetic polymers such as acrylamide (AAm), acrylic acid (AAc), and copolymers of AAm have been utilized to form hydrogels and entrap macronutrients like nitrogen, phosphorus, and potassium (NPK). For example, Liu et al. (2007) reported the application of AAc hydrogel formed from N,N′-methylene bisacrylamide as a crosslinker and ammonium persulfate as an initiator in the presence of urea. This hydrogel exhibited a slow-release profile, delivering approximately 80% of the entrapped nitrogen over a period of approximately 28 days [8]. The same material has been used to trap phosphorous, using KH_2_PO_4_ as the source, demonstrating a controlled release of this nutrient [9]. However, despite the effectiveness of hydrogels formed using synthetic polymers in facilitating slow nutrient release, their high production costs and low biodegradability make them unsuitable for agriculture applications [10]. To address these drawbacks, researchers have explored combining synthetic polymers with naturals biopolymers (e.g., cellulose, starch, and chitosan) and clays (e.g., bentonite, kaolin, and montmorillonite) to enhance the properties of hydrogels and reduces production costs [11,12].

Biopolymers, which are biodegradable, non-toxic, and biocompatible materials derived from natural sources, show a robust alternative for forming hydrogels. They contribute to reducing biomass waste and protecting the environment [13]. For instance, a carboxymethyl starch-g-polyacrylamide hydrogel was developed and used for the slow release of phosphorous. This hydrogel delivered approximately 87% of the nutrient over 30 days, highlighting its potential as a material for controlled nutrient supply [14]. However, the literature has explored only a limited range of biopolymers, such as cellulose, chitosan, and starch, to enhance the biodegradability of hydrogels. Starch is one of the most abundant polysaccharides worldwide, found in high concentrations in vegetables and cereals. It is composed of a co-polymer chain consisting of amylose (AM) and amylopectin (AP), typically comprising approximately 20–30% AM and 70–80% AP. Additionally, starch is also a biodegradable, biocompatible, and non-toxic biopolymer [15,16]. Several crosslinking compounds have been reported for producing starch hydrogels, such as glutaraldehyde, epichlorohydrin, and sodium hypophosphite. However, their high cost and negative environmental impacts have limited their use in agriculture applications. Hence, to achieve an eco-friendly and cost-effective hydrogel material, citric acid (2-hydroxypropane-1,2,3 tricarboxylic acid, C_6_H_8_O_7_) was investigated as a crosslinking agent for cassava starch biopolymer. The crosslinking reaction was monitored using ATR-FITR measurements, and the swelling behavior and water retention properties were determined and compared. Additionally, the materials were employed for the entrapment of potassium and nitrogen, and their slow-release profiles were assessed. This work provides valuable insights into the production of cassava starch–citric acid hydrogels, which have the potential to enhance the macronutrient release while mitigating the negative environmental impacts of the fertilizer.

## 2. Results and Discussion

### 2.1. Hydrogel Characterization

**Figure 1:**
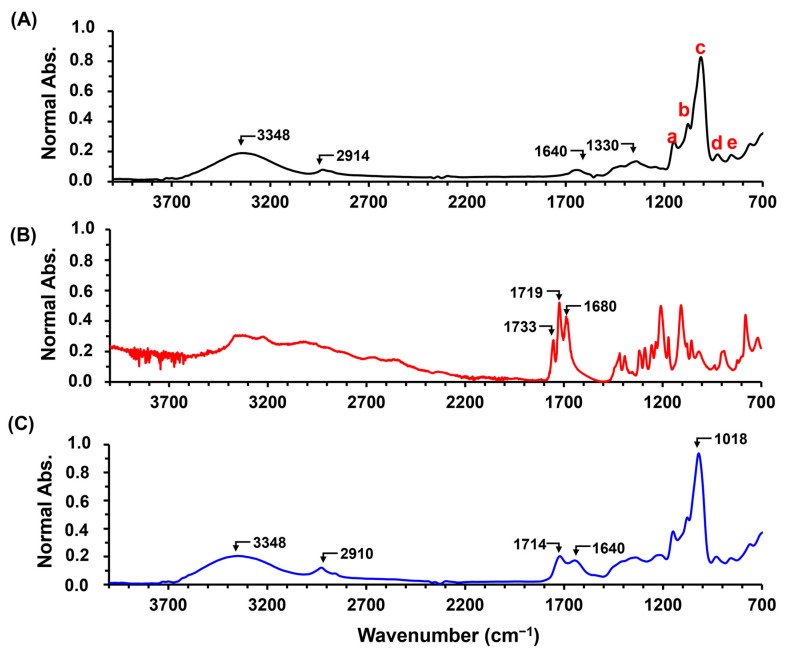
ATR-FITR spectra of CA and hydrogels: (**A**) AGCY, (**B**) CA, and (**C**) AGCY-CA(10). In spectrum A, bands related to monomeric units are identified with a, b, c, d and e (see details in Section 2.1).

The crosslinking reaction between cassava starch (AGCY) and citric acid (CA) to form starch hydrogel (AGCY-CA(10)) was performed using a concentration of 15% and 10% *w*/*w* of AGCY and CA, respectively. Figure 1 shows the main bands in the ATR-FITR spectrum of AGCY, CA, and AGCY-CA(10). In cassava starch (Figure 1A), a band around 3348 cm^−1^ can be observed and is attributed to the stretching of the hydroxyl groups. The band at 1640 cm⁻^1^, indicating the presence of water in the biopolymer, suggests the high hydrophilicity of cassava starch [17]. Additionally, a band around 2914 cm^−1^ appears, corresponding to the stretching of C–H groups in the biopolymer chain. Bellow 1700 cm^−1^, bands related to AM and AP units are observed: the band at 1330 cm^−1^ is attributed to the symmetric deformation and scissoring vibration of the CH_2_ groups in the glucose units. Furthermore, bands a (1139 cm^−1^) and b (1078 cm^−1^) correspond to the C–O stretching, while bands c (1016 cm^−1^), d (918 cm^−1^), and e (846 cm^−1^) are attributed to the C–O–C ring vibration in the monomer units [17,18].

**Figure 2:**
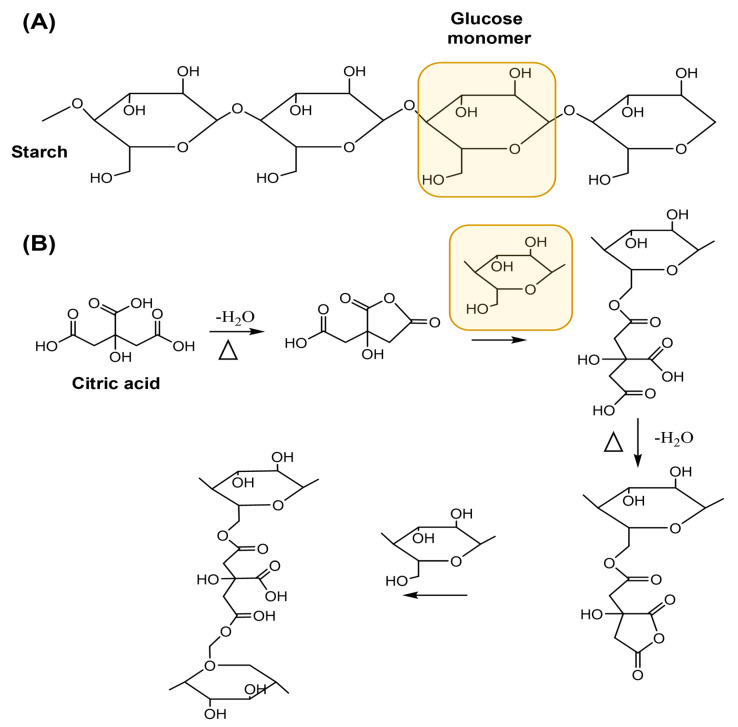
(**A**) Starch structure. (**B**) Mechanism of crosslinking reaction between CA and starch.

On the other hand, the ATR-FITR spectrum of CA (Figure 1B) exhibits three bands between 1700 cm^−1^ and 1600 cm^−1^, attributed to the stretching of the carboxylic (C=O) groups of CA [19]. In the ATR-FITR spectrum of the AGCY-CA(10) hydrogel, similar bands were observed in the fingerprint region, attributed to the vibration of the AM and AP units. Additionally, a band appears at 1714 cm^−1^ (Figure 1C), confirming the crosslinking reaction between the carboxyl groups of the CA and the hydroxyl groups of the cassava starch to form ester groups. Due to the steric hindrance, the hydroxyl at position C6 of the starch is more reactive than the hydroxyl at positions C2 and C3 (Figure 2) [20,21]. During the production of AGCY-CA(10) hydrogel, CA is converted to its anhydride, forming ester bonds with the hydroxyl groups of anhydroglucoses from neighboring chains of cassava starch [22]. This process results in greater stabilization of the starch structure in the material. These results are consistent with other studies on starch esterification reactions with CA [23,24].

Furthermore, the intense bands around 1640 cm^−1^ indicate the presence of water in the formed material. The higher intensity of this band compared to the cassava starch spectrum suggests a significant presence of water, likely entrapped within the hydrogel’s polymeric matrix. This band could also be attributed to the carbonyl groups of the citric acid that are not crosslinked with starch [25]. Incomplete crosslinking can leave citrate groups available to interact electrostatically with ions such as K^+^ and NH4^+^, promoting favorable interactions to retain theses fertilizer ions in the polymeric network and enable slow-release behavior. Moreover, CA is recognized as a safe food additive and is considered safe for the environment and human health [26,27]. This is an important characteristic for materials applied in nutrient release in soils to enhance agricultural productivity, as it does not negatively impact the environment. Additionally, starch esterification with CA increases starch resistance to amylolysis (i.e., transformation of starch into sugar by the effect of acids or enzymes) [28]. Consequently, it could increase degradation resistance in soils, prolonging the time for fertilizer release, and inhibiting nutrient losses through processes such as leaching, runoff, volatilization, and lixiviation.

**Figure 3:**
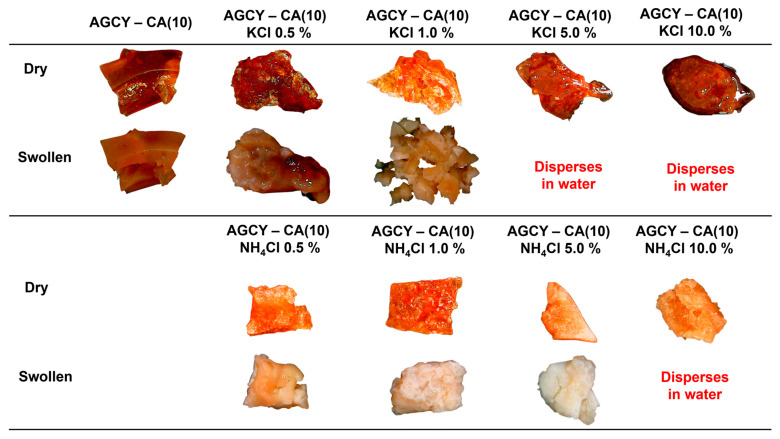
Digital photos of AGCY-CA(10) with and without several KCl and NH_4_Cl concentrations (0.5 to 10.0% *w*/*w*) before and after water-absorbing process.

**Figure 4:**
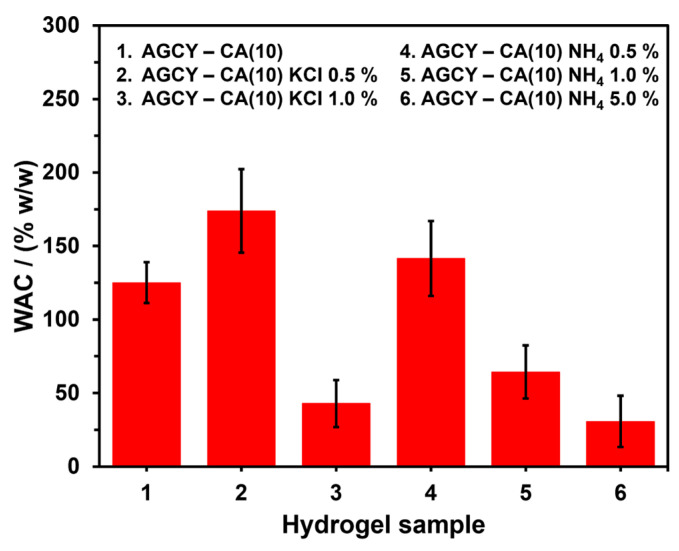
WAC of the AGCY-CA(10) hydrogel with and without KCl and NH_4_Cl in water.

To facilitate the slow delivery of nutrients, hydrogel was applied using different concentrations (0.5 to 1.0% *w*/*w*) of KCl and NH_4_Cl as sources of potassium and nitrogen, respectively (Figure 3). The material AGCY-CA(10) without nutrients exhibited a reddish-brown color and a dry, rigid appearance, which remained after water absorption (swollen), indicating a high degree of crosslinking and a lower WAC. These findings are congruent with the results reported by Mei et al. (2015), who observed that citrate-crosslinked starch granules showed lower WAC in comparison with native starch. This reduction in WAC is attributed to changes in the AM and AP structures, which hinder granule swelling [29]. However, upon incorporating nutrient salt sources, the materials became more malleable as the nutrient concentration increased. Even at concentrations of 5.0% and 10.0% KCl, the materials did not form a rigid structure and dispersed in water after immersion. In contrast, the materials formed with 0.5% and 1.0% KCl maintained their structure after water absorption. On the other hand, when the hydrogel was formed with NH_4_Cl, all the materials maintained their structure after water absorption, except for the hydrogel formed with 10.0% NH_4_Cl, which dispersed in water. Additionally, as the NH_4_Cl concentration increased, the material became softer after water absorption, suggesting that the addition of nutrient salts inhibits starch crosslinking or modifies the starch’s chemical structure. This is further supported by the WAC results, as the increase in nutrient source salts led to a decrease in WAC (Figure 4), attributed to a reduction in the crosslinking reaction, which reduces the material’s capacity to absorb water. It is important to highlight that the AGCY-CA(10) hydrogel exhibited a lower WAC (125.1 ± 13.9%) compared to the materials formed with 0.5% KCl (173.9 ± 28.4%) and 0.5% NH_4_Cl (141.5 ± 25.5%).

**Figure 5:**
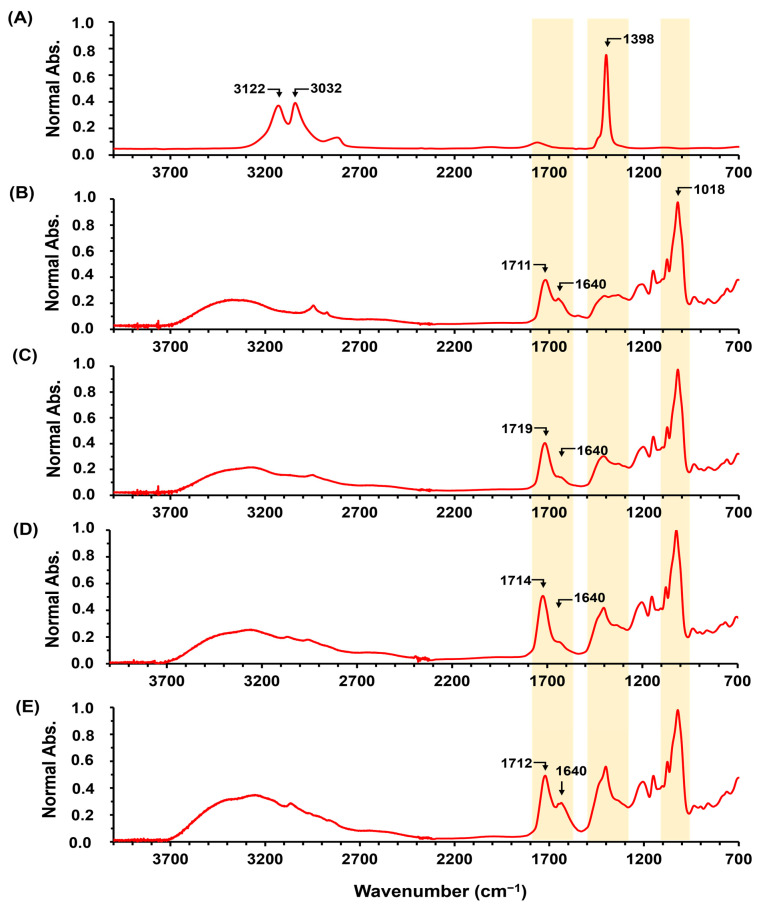
ATR-FITR spectra of fertilizer and hydrogels: (**A**) NH_4_Cl and AGCY-CA(10) with several concentrations of NH_4_Cl: (**B**) 0.5%, (**C**) 1.0%, (**D**) 5.0%, and (**E**) 10.0%.

**Figure 6:**
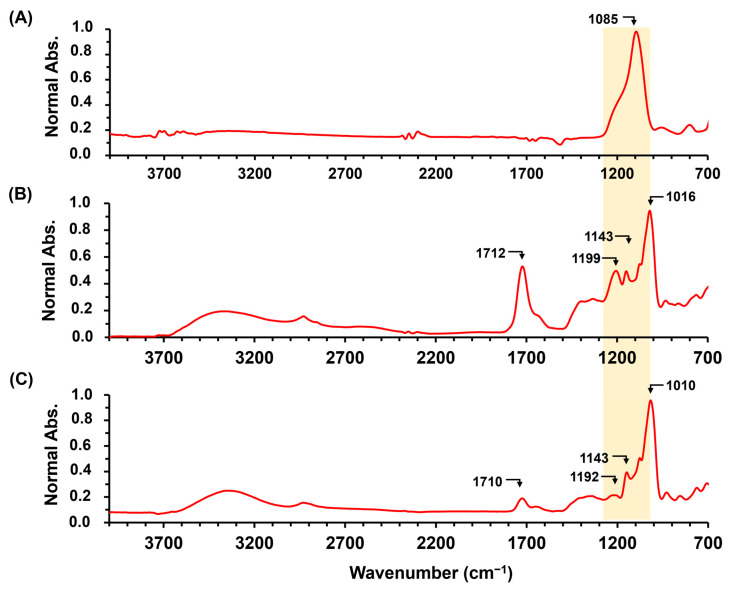
ATR-FITR spectra of (**A**) KCl and hydrogel AGCY-CA(10) with several concentrations of KCl: (**B**) 0.5% and (**C**) 1.0%.

ATR-FTIR spectra of hydrogels loaded with fertilizers are shown in Figure 5 and Figure 6. The spectrum of NH_4_Cl exhibited three principal bands at 3122, 3032, and 1398 cm^−1^ attributed to the NH_4_^+^ stretching vibrations [30]. The intensity of the band at 1398 cm^−1^ increased with a higher NH_4_Cl concentration in the hydrogels. Furthermore, the peak associated with ester formation, reaching its maximum intensity between 1710 and 1720 cm⁻^1^, suggests that the presence of low concentrations of fertilizer did not significantly impact the crosslinking of cassava starch. However, based on both digital photos of the materials and ATR-FTIR results, it is evident that despite observing a band at 1712 cm⁻^1^ in the material containing 10% NH_4_Cl, the degree of esterification was not substantial. This is evidenced by the material dispersing in water after 24 h.

Furthermore, all AGCY-CA materials with fertilizer showed the same band profiles of glucose units in the starch, demonstrating that there were no significant modifications in the structure of amylose and amylopectin during material formation. On the other hand, KCl exhibits an intense band at 1085 cm^−1^, which is likely attributed to the Si–O stretching of impurities in the fertilizer used. In the hydrogel AGCY-CA(10) with KCl 0.5%, a band around 1712 cm^−1^ is observed, indicating AGCY-CA esterification. However, this band notably decreases with 1.0% KCl, suggesting that the fertilizer reduces the efficiency of the crosslinking reaction. This finding is consistent with the analysis using digital photos of the dry and swollen material, where an increase in KCl concentration leads to the reduced physical stability of the material. Even at concentrations higher than 5.0%, the material does not swell and disperses in water.

During the material formation, three processes are involved: (i) gelatinization, (ii) crosslinking reaction, and (iii) retrogradation. In the system, the fertilizer is added after cassava starch gelatinization; therefore, the fertilizer is expected to affect steps (ii) and (iii) of the material formation process, limiting crosslinking and/or the association and hydrogen bonding between the linear chains of AM and AP. It is known that cations can significantly influence starch retrogradation, particularly bivalent cations such as Ca^2+^ and Mg^2+^, as these ions affect the hydrogen bonds in water–starch and starch–starch systems [31,32]. The negative effect on retrogradation occurs because the interaction between the cation and starch hydroxyl groups causes the starch to become negatively charged, resulting in a positive charge in the water phase. This creates a potential difference known as the Donnan potential [33]. Therefore, it is likely that potassium ions induce a negative charge on the starch, leading to intramolecular repulsive forces within the starch and affecting the starch–starch association. This explains why, at high concentrations of salt, the material does not form a rigid structure. Interestingly, this effect was also observed with ammonium salts, although it only occurred at fertilizer salt concentrations higher than 5.0%.

### 2.2. Release Studies In Vitro of Potassium and Nitrogen

**Figure 7:**
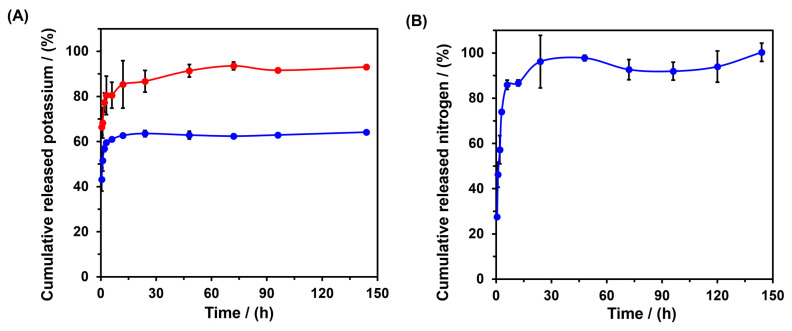
Cumulative nutrient release profiles from hydrogel AGCY-CA(10) with (**A**) KCl 0.5% (•) and 1.0% (•) and (**B**) NH_4_Cl 1.0% (•).

The potassium and nitrogen release profiles from hydrogels AGCY-CA(10) were evaluated using materials with 0.5 and 1.0% KCl, and the material with 5.0% NH_4_Cl (Figure 7A,B). The in vitro release profile of potassium from the cassava starch hydrogel showed an initial burst release, with approximately 80% and 60% of the entrapped nutrient released in the first 6 h for the materials formed with 0.5% and 1.0% KCl, respectively. Subsequently, for the systems with low KCl concentrations, the nutrient release increased gradually, reaching almost 90% of the entrapped potassium after approximately 60 h. After that, the released potassium levels remained relatively constant with increasing time. This behavior may be attributed to the rapid dissolution and diffusion of potassium at the beginning of the swelling process. However, for the material with 1.0% KCl, the release did not exceed 64% of the entrapped potassium. This suggests that the higher KCl concentration led to multiple desorption–adsorption steps, which contributed to maintaining a significant proportion of entrapped potassium (approximately 34%). On the other hand, when nitrogen was entrapped, the percentage of released nutrients increased rapidly from 27.4% to 96.2% as time increased from 30 min to 24 h, indicating its ability to gradually release the nutrient within the first 24 h. The rapid release of ammonium ions could be attributed to their instantaneous migration to the release medium when the sample is immersed. A similar release behavior was observed by León et al. (2019), who found that starch oxidate hydrogel released 90% of the entrapped ammonium within the first 4 h of the release experiment [34].

Four kinetic models (zero-order, first-order, Higuchi, and power-law) were considered to evaluate the mechanism of nutrient release from the materials formed. Table 1 presents the calculated kinetic parameters obtained by fitting the experimental data. Among the models applied to the release data, the power-law model showed the highest regression coefficients (R^2^) for both nutrients.

This mathematical model describes solute diffusion from a matrix with different geometric dimensions and porous systems, allowing for inference of the mechanisms associated with nutrient release based on the release exponent (n) value: n = 0.45 corresponds to a Fickian diffusion mechanism or solute release that is diffusion-controlled; n = 0.45–0.89 corresponds to non-Fickian transport (anomalous diffusion) or solute release that is both diffusion-controlled and erosion-controlled; n = 0.89 relates to a case II mechanism (polymer relaxation or swelling-controlled); and n > 0.89 relates to a super case II mechanism (transport or solute release that is erosion-controlled) [35,36,37]. According to the power-law model, the release of nutrients occurs through the diffusion of nutrients, which are rapidly delivered upon swelling of the hydrogel AGCY-CA(10). When the polymeric material swells, it induces macromolecular relaxation of the biopolymer, resulting in a rubbery state. In this state, solute diffusion into the aqueous medium generally occurs. However, in the case where the water penetration rate is much slower than the polymer chain relaxation rate, the value of “n” can be less than 0.5, as observed in the tested materials. This phenomenon is still considered Fickian diffusion and is referred to as “Less-Fickian”. For the hydrogels studied, the release of potassium (n = 0.5) and nitrogen (n = 0.55) follows a Less-Fickian and anomalous diffusion pattern, respectively [36,38]. This means that the release process of potassium is primarily dependent on the rate of water penetration, where the biopolymers have high mobility, and water easily penetrates the rubbery network, promoting rapid diffusion of the nutrient out of the matrix. Conversely, for nitrogen, the material exhibits high mobility, allowing for the rapid penetration of water into the biopolymer matrix [34].

### 2.3. Production and Application of Economic Starch—CA Hydrogel

**Figure 8:**
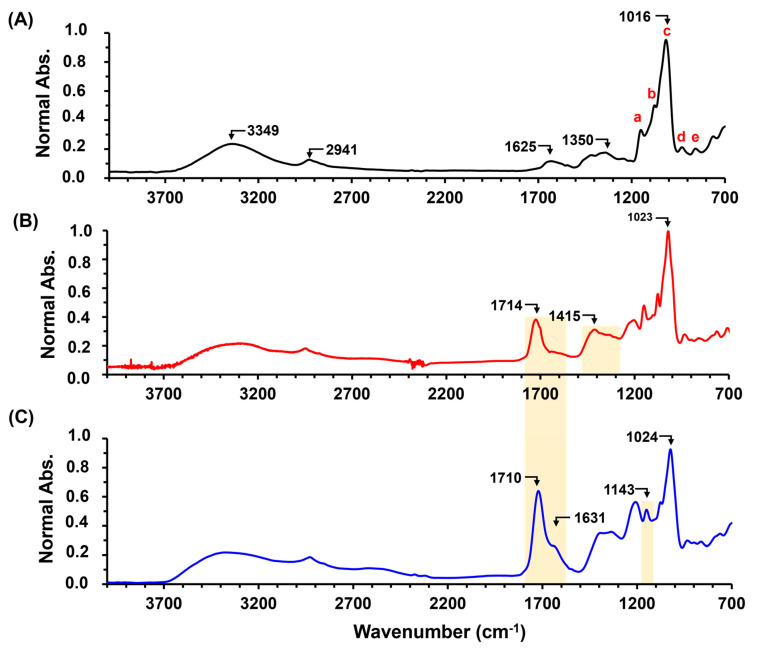
ATR-FITR spectra of (**A**) AYD, (**B**) AYD-CA(10)-NH_4_Cl 1.0%, and (**C**) AYD-CA(10)-KCl 1%. In spectrum A, bands related to monomeric units are identified with a, b, c, d and e (see details in Section 2.1).

CA is an economical raw material and is considered a green chemical compared to other crosslinking agents reported in the literature for starch, such as glutaraldehyde, epichlorohydrin, boric acid, and sodium hypophosphite. In order to decrease production costs, cassava with peel (AYD) was milled to replace the cassava starch used in the previous experiments and applied in the entrapment of N and K macronutrients. Figure 8A shows the ATR-FTIR spectra of AYD, AYD-CA(10) with NH_4_Cl 1.0% (Figure 8B), and KCl 1.0% (Figure 8C). The AYD material exhibits similar bands to those reported for cassava starch, especially in the fingerprint region (600–1400 cm^−1^), indicating the presence of a high quantity of amylose and amylopectin units in the material. This allows for its application in forming hydrogels, eliminating the need for starch extraction. Additionally, both spectra of the formed material show an intense band around 1700 cm^−1^, indicating material crosslinking and the successful production of the economical hydrogel. These results are important because they demonstrate that it is not necessary to extract cassava starch in order to obtain a hydrogel with the capacity to reduce nutrient release in soils.

**Figure 9:**
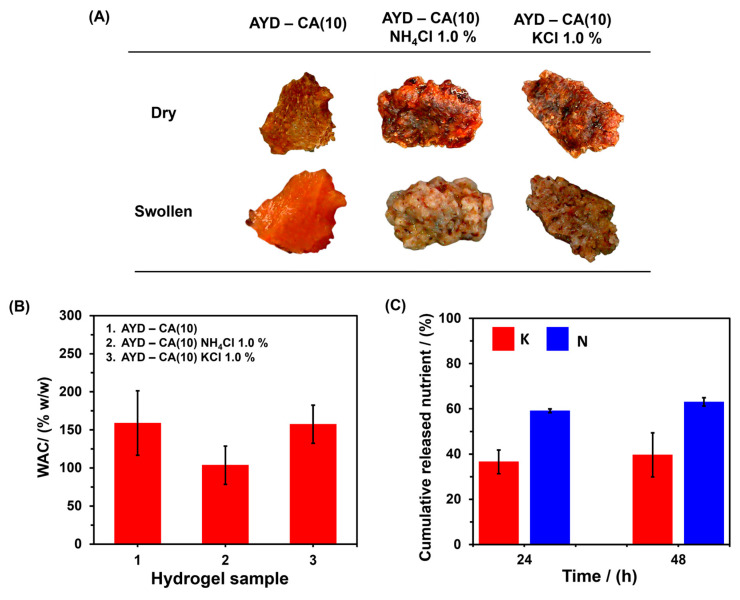
AYD-CA(10) with and without KCl (1.0%) and NH_4_Cl (1.0%): (**A**) Digital photos before and after water-absorbing process, (**B**) WAC in water, and (**C**) Potassium and nitrogen cumulative nutrient release during 24 h and 48 h.

The material AYD-CA(10), with and without fertilizer, exhibits a rigid structure both before and after immersion in water for 24 h, maintaining its physical integrity without dispersion in the swelling medium (Figure 9A). However, a heterogeneous surface can be observed due to the presence of cassava peel. Additionally, the materials have the ability to absorb water, with AYD-CA(10) showing a WAC of 183.4 ± 0.6%, which is higher than that of AGCY-CA(10). This effect is likely caused by the heterogeneity of the cassava sample, which reduces the crosslinking points in the material and allows for greater penetration of water into the biopolymer network. Furthermore, similar to the AGCY-CA(10) materials, the addition of nutrients decreases the WAC of the material AYD (Figure 9B), resulting in a WAC of 117.9 ± 3.7% for the material with NH_4_Cl and 157.4 ± 25.0% for the material with KCl. This is likely because the fertilizers reduce the efficiency of crosslinking, decreasing the formation of cavities in the material for water entrapment. Moreover, the application of the material for potassium release shows similar behavior to AGCY-CA(10)-KCl 1.0%, delivering approximately 40% of the entrapped nutrient in 48 h (Figure 9C). However, for nitrogen, the material exhibits improved nutrient retention, delivering approximately 60% of the entrapped nutrient. This indicates that the material AYD-CA(10) is economical and enhances the capacity to retain nutrients compared to AGCY-CA(10). The above is a result of raw materials and methodology; for instance, hydrogels are produced from cassava starch with peel, which eliminates the need for AGCY and reduces the cost of hydrogel production, as AGCY is obtained from AYD and requires different physical processes. Additionally, after 48 h of nutrient release, AYD-CA(10) retains nearly 60% K and 40% N. These values are higher than those for AGYD-CA(10), which retains 40% K and 3% N. Thus, it has the potential to facilitate nutrient applications in various agricultural practices, reducing the environmental impacts of conventional application methods.

## 3. Conclusions

Cassava starch hydrogels were successfully prepared through a crosslinking reaction between starch and CA in an aqueous solution. This methodology is considered simple and economical. The hydrogel material was then utilized to entrap potassium and nitrogen, facilitating the controlled release of these macronutrients. The ATR-FTIR analysis demonstrated that the incorporation of nutrient sources, such as KCl and NH_4_Cl, influenced the formation of the hydrogel material. Higher concentrations of these salts were found to reduce the efficiency of crosslinking. Additionally, the presence of nutrients resulted in a decrease in the WAC of the hydrogel materials. The cassava starch hydrogel exhibited the ability to effectively entrap and release the tested macronutrients. The release mechanism followed a Less-Fickian diffusion for potassium and anomalous diffusion for nitrogen. This indicates that the release of potassium is mainly dependent on the water penetration rate, allowing for rapid diffusion outside the rubbery network of the hydrogel. On the other hand, the release of nitrogen is influenced by the high mobility of the hydrogel, which facilitates the penetration of water into the biopolymer matrix. Furthermore, the production of hydrogels using cassava with peel material, which was previously milled, was successfully demonstrated. This approach aimed to reduce production costs and eliminate the need for starch extraction. The resulting materials exhibited improved nitrogen retention compared to cassava starch hydrogels. This economic hydrogel material offers the advantage of being suitable for production in farms with limited infrastructure and can be applied in the agroindustry to minimize the negative environmental impacts of nutrient application.

## 4. Materials and Methods

### 4.1. Materials

Cassava and cassava starch (AGCY) were acquired from a local market in Cali, Colombia. Citric acid (CA) (C₆H₈O₇), sodium hydroxide (NaOH), formaldehyde (CH₂O), and potassium hydrogen phthalate (KHC₈H₄O₄) were purchased from Sigma-Aldrich (St. Louis, MA, USA). Potassium chloride (KCl) and ammonium chloride (NH_4_Cl) were obtained from a fertilizer warehouse in Cali, Colombia.

### 4.2. Preparation of Starch-CA Hydrogels

The AGCY-CA hydrogels were prepared using the following procedure: A specific amount of AGCY that would achieve a starch concentration of 15% *w*/*w* was mixed with distilled water and stirred. The mixture was then heated at 75 °C for 30 min to facilitate the gelatinization of starch. After that, a predetermined quantity of CA was added to obtain a CA concentration of 10% *w*/*w*. The mixture was continuously stirred for 1 h at 75 °C, and the resulting material was dried at 50 °C for 72 h. On the other hand, to form cost-effective hydrogels, the cassava was washed and milled to obtain a cassava flour (AYD). The AYD-CA hydrogels were then formed using the same methodology described above.

### 4.3. Swelling Studies

The WAC was evaluated using the tea bag method described by Palencia et al. (2017) [39]. A known mass of dried hydrogel was placed inside a pre-weighed tea bag and submerged in distilled water for 24 h. Subsequently, tea bag containing the hydrogel was suspended for 15 min to allow the excess water to drain off. The tea bag, along with the sample, was weighed to determine the WAC using Equation 1. As a control, a blank experiment was conducted using only the empty tea bag.

(1) $$WAC=\left(\frac{w_{2}-(w_{1}+w_{0})}{w_{1}}\right)-{WAC}_{0}$$

where $w_{0}$, $w_{1}$, and $w_{2}$ are the masses of the tea bag, the sample, and the sample tea-bag system, respectively, and ${WAC}_{0}$ is the correction of WAC given the results of blank experiment. These experiments were conducted in triplicate to ensure accuracy and reproducibility of the results.

### 4.4. Physicochemical and Morphological Characterizations of the Hydrogels

Fourier-transformed infrared spectroscopy measurements using attenuated total reflectance (ATR-FTIR) were conducted on the hydrogels to verify the esterification reaction. The measurements were performed using an IR-Affinity-1 instrument (Shimadzu, Kyoto, Japan). Data acquisition was carried out in the range of 4000 to 700 cm^−1^ at a rate of 2 cm^−1^ per data point, with 20 scans recorded for each sample. The measurement was performed in triplicate. In addition to spectroscopy analysis, digital photographs were taken of the hydrogel materials before and after immersion in water. These photographs were captured using a Flexible Digital Electronic Microscope (Suzhou Jingtong Instrument Co., Ltd., Suzhou, China) to visually assess the changes in the samples upon water exposure.

### 4.5. Preparation of AGCY-CA with Potassium and Nitrogen Nutrients Entrapped

AGCY-CA and AYD-CA materials were prepared as described earlier in the “Preparation of starch-CA hydrogels” section. In addition, KCl and NH_4_Cl were independently added to form Y-CA-KCl-X and Y-CA-NH_4_Cl-X materials, where Y represents the starch source (Y = AGCY or AYD) and X represents the salt concentration (0.5% or 1.0% or 5.0% or 10.0% *w*/*w*). For AYD-CA, both KCl and NH_4_Cl were incorporated at a concentration of 1.0% *w*/*w*. To determine the concentration of potassium and nitrogen, the following methodology was employed: Potassium quantification was carried out using an atomic absorption spectrophotometer (AA-6300, Shimadzu, Kyoto, Japan) following the procedure outlined in the Colombian Institute of Technical Standards and Certification (NTC 202) [40]. The sample was first digested in a mixture of HCl/HNO_3_ with a 2:1 ratio, followed by filtration using quantitative filter paper. A potassium curve was prepared using concentrations ranging from 0.2 to 1.0 ppm in deionized water with the addition of 1.0% lanthanum chloride (LaCl_3_).

On the other hand, nitrogen concentration was determined by titration of ammonium in the fertilizer sample, using the methodology outlined in the Colombian Institute of Technical Standards and Certification (NTC 5268) [41]. First, the sample was dissolved in deionized water, followed by the addition of 40% *v*/*v* formaldehyde and 30 mL of water. The sample was titrated with NaOH in the presence of phenolphthalein. NaOH solution was standardized using KHC₈H₄O₄, which was previously dried at 98 °C for 4 h. The amount of NaOH used in the titration provided the ammonium content in the sample, with a 1:1 molar ratio, allowing the determination of the nitrogen concentration.

### 4.6. Nutrients Release Kinetics—In Vitro Study

To evaluate the release of nutrients from AGCY-CA(10%) and AYD-CA(10%) hydrogels, approximately 1.0 g of each hydrogel with entrapped nutrients was placed in tea bags. Release experiments were conducted by continuously agitating the samples at 100 rpm in deionized water as the release medium. At specified time intervals over a period of six days, 2.0 mL aliquots of the resulting solution were withdrawn and immediately replaced with 2.0 mL of fresh medium. The experiments were performed in triplicate to ensure accuracy and reproducibility. The concentrations of potassium and nitrogen in the withdrawn solution samples were determined using atomic absorption spectroscopy and titration methods, respectively, as described in the previous sections. For the nitrogen quantification, a titration blank was performed using 1.0 mL of the sample solution with NaOH, employing phenolphthalein as an indicator, without the addition of formaldehyde. To analyze the release mechanism of the nutrients, four kinetic models were employed: zero-order, first-order, Higuchi, and power-law. These models can be represented by the following equations (Equations (2)–(5), respectively):

(2) $$\mathrm{Zero}\text{-}\mathrm{order}\;\mathrm{model}:\;M_{t}/M_{\infty }=k_{0}t$$

(3) $$\mathrm{First}\text{-}\mathrm{order}\;\mathrm{model}:\;M_{t}/M_{\infty }=1-e^{\left(-k_{1}t\right)}$$

(4) $$\mathrm{Higuchi}\;\mathrm{model}:\;M_{t}/M_{\infty }=k_{H}t^{\frac{1}{2}}$$

(5) $$\mathrm{Power}\text{-}\mathrm{law}\;\mathrm{model}:\;M_{t}/M_{\infty }=kt^{n}$$

where $M_{t}/M_{\infty }$ represents the fraction of nutrient released at time t. $k_{0}$, $k_{1}$, $k_{H}$, and k represent the zero-order release constant, first-order release constant, Higuchi constant, and power-law constant, respectively. The release constants and model parameters were determined by fitting the experimental release data [42,43].

## Figures and Tables

**Table 1:** Release kinetics parameters for potassium and nitrogen release from the AGCY-CA(10) hydrogel.

	Mathematical Models								
Fertilizer/Concentration	Zero-Order		First-Order		Higuchi		Power-Law		
	k_0_ ($1\times 10^{-2}\;h^{-1}$)	R^2^	k_1_ ($1\times 10^{-1}\;h^{-1}$)	R^2^	k_H_ ($1\times 10^{-1}\;h^{-1}$)	R^2^	K ($1\times 10^{-1}\;h^{-1}$)	R^2^	n
KCl/0.5%	3.4	0.7747	7.0	0.8157	1.0	0.9005	7.81	0.9447	0.13
KCl/1.0%	1.7	0.2154	1.5	0.2662	0.7	0.3958	1.49	0.995	0.14
NH_4_Cl/5%	1.2	0.8564	2.7	0.8907	3.92	0.8986	4.21	0.9791	0.55

k_0_, k_1_ “k_H_”, and “k” represent the apparent release rate constants of the respective mathematical models, and n is the release exponent of the power-law model.

## Data Availability

Data are contained within the article.
